# Supplementary figures and images for: Transcriptomic Analysis Reveals the Impact of the Biopesticide Metarhizium anisopliae on the Immune System of Major Workers in Solenopsis invicta
Source: Insects. 2023 Aug 11;14(8):701. doi: 10.3390/insects14080701 (PMC10455567; doi:10.3390/insects14080701)

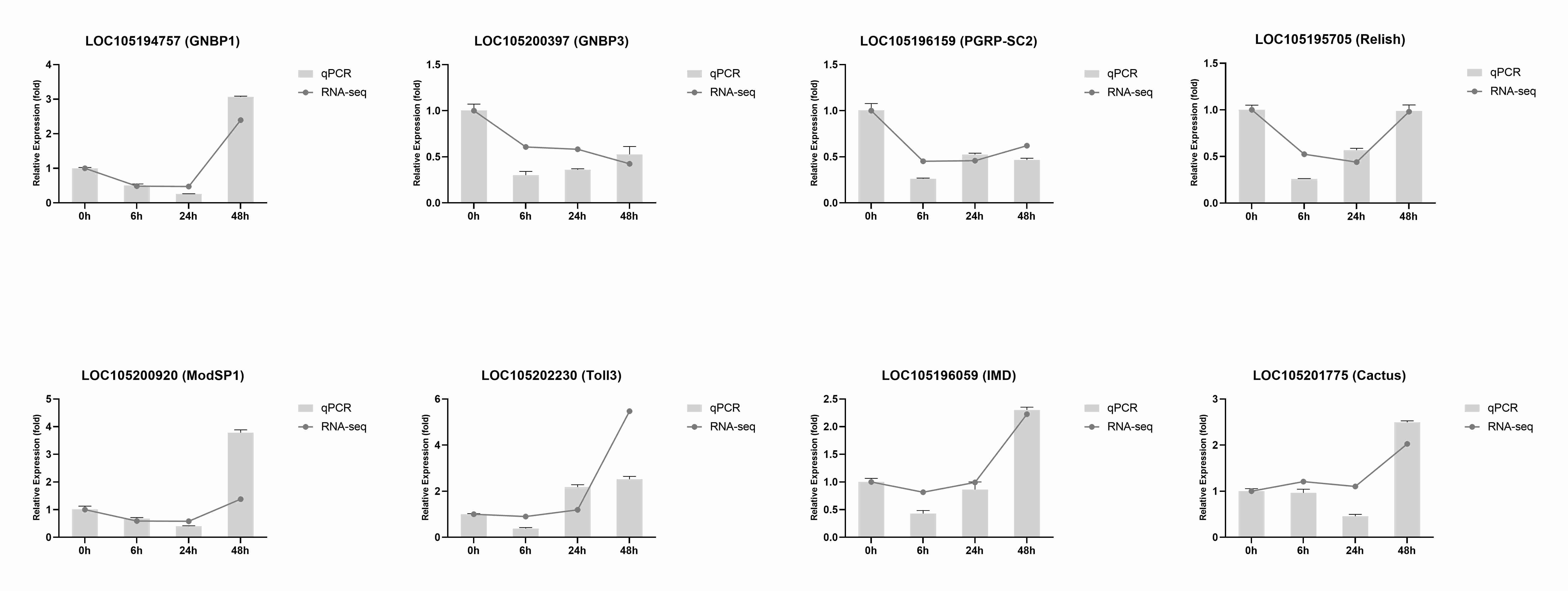

Supplement: Supplementary file 1 [file insects-14-00701-s001.zip › Figure S1 Validation of Transcriptome via RT-qPCR.jpg]
